# Prenatal Endotoxin Exposure Induces Fetal and Neonatal Renal Inflammation via Innate and Th1 Immune Activation in Preterm Pigs

**DOI:** 10.3389/fimmu.2020.565484

**Published:** 2020-09-30

**Authors:** Tik Muk, Ping-Ping Jiang, Allan Stensballe, Kerstin Skovgaard, Per Torp Sangild, Duc Ninh Nguyen

**Affiliations:** ^1^Section for Comparative Paediatrics and Nutrition, Department of Veterinary and Animal Sciences, University of Copenhagen, Copenhagen, Denmark; ^2^School of Public Health, Sun Yat-sen University, Guangzhou, China; ^3^Department of Health Science and Technology, Aalborg University, Aalborg, Denmark; ^4^Department of Biotechnology and Biomedicine, Technical University of Denmark, Copenhagen, Denmark; ^5^Department of Paediatrics, Odense University Hospital, Odense, Denmark

**Keywords:** intrauterine bacterial infection, chorioamnionitis, plasma proteomics, renal inflammation, acute kidney injury, immune activation

## Abstract

Chorioamnionitis (CA) predisposes to preterm birth and affects the fetal mucosal surfaces (i.e., gut, lungs, and skin) via intra-amniotic (IA) inflammation, thereby accentuating the proinflammatory status in newborn preterm infants. It is not known if CA may affect more distant organs, such as the kidneys, before and after preterm birth. Using preterm pigs as a model for preterm infants, we investigated the impact of CA on fetal and neonatal renal status and underlying mechanisms. Fetal pigs received an IA dose of lipopolysaccharide (LPS), were delivered preterm by cesarean section 3 days later (90% gestation), and compared with controls (CON) at birth and at postnatal day 5. Plasma proteome and inflammatory targets in kidney tissues were evaluated. IA LPS-exposed pigs showed inflammation of fetal membranes, higher fetal plasma creatinine, and neonatal urinary microalbumin levels, indicating renal dysfunction. At birth, plasma proteomics revealed LPS effects on proteins associated with renal inflammation (up-regulated LRG1, down-regulated ICA, and ACE). Kidney tissues of LPS pigs at birth also showed increased levels of kidney injury markers (*LRG1*, *KIM1*, *NGLA*, *HIF1A*, and *CASP3*), elevated molecular traits related to innate immune activation (infiltrated MPO^+^ cells, complement molecules, oxidative stress, *TLR2*, *TLR4, S100A9*, *LTF*, and *LYZ*), and Th1 responses (CD3^+^ cells, ratios of *IFNG/IL4*, and *TBET/GATA3*). Unlike in plasma, innate and adaptive immune responses in kidney tissues of LPS pigs persisted to postnatal day 5. We conclude that prenatal endotoxin exposure induces fetal and postnatal renal inflammation in preterm pigs with both innate and adaptive immune activation, partly explaining the potential increased risks of kidney injury in preterm infants born with CA.

## Introduction

Preterm birth (before 37 weeks of gestation, 15 million cases per year, and ∼10% of all pregnancies) is a global health problem and a leading cause of infant mortality and morbidity (1, 2). A majority of preterm births (40–70%) are caused by chorioamnionitis (CA, inflammation of the fetal membranes), which is often derived from bacterial infections in the lower genital tract and amniotic fluid (3–5). CA is often associated with multiple neonatal complications after preterm birth, including necrotizing enterocolitis (NEC), bronchopulmonary dysplasia, periventricular leukomalacia, and sepsis, depending on the location, timing, and severity of maternal inflammation (6). Despite these associations, it remains unclear how CA interacts with reduced gestational age at birth (preterm birth) to affect infant organs, both at birth and later.

Due to the limited access to biological samples in infants, animal models are essential to better understand the underlying organ-specific, pathophysiological mechanisms of prenatal insults. Intra-amniotic (IA) administration of lipopolysaccharide (LPS) has been used to induce CA in rhesus macaques, sheep, and mice (7–10). However, the postnatal effects of prenatal insults in these models are largely unknown, partly due to difficulties in rearing preterm animals. Exposure to IA LPS or microbes often elevates levels of immune cells and pro-inflammatory cytokines in the amniotic fluid, which are thought first to interact with fetal mucosal surfaces (e.g., gut, lung, and skin) to evoke local tissue inflammation, and later the inflammatory signals may or may not extend to the circulation and other internal organs (11, 12). In rodents, lambs, and pigs, CA effects on fetal lung and gut inflammation (via neutrophil and/or macrophage infiltration and TLR signaling modulation) (13–19) as well as on fetal acute brain injuries (20, 21) have well been investigated. In contrast, the effects on other internal organs, both at birth and in postnatal periods, remain elusive.

Neonatal kidney inflammation and acute kidney injury (AKI) are under-recognized neonatal conditions with challenging diagnosis due to the lack of reliable diagnostic biomarkers (22). As nephrogenesis mainly occurs in the last trimester of human pregnancy (23) and *extra-uterine* environment seems not optimal for glomerular development (24), preterm infants, especially those born before 28 weeks of gestation, may be at high risks of developing AKI. In fact, ∼50% of preterm infants with extremely low birth weight (<1000 *g*) are diagnosed with early-onset AKI and AKI is also associated with increased mortality (25–29). Neonatal AKI can also be induced by fetal distress and postnatal exposure to hypotension, sepsis, and nephrotoxic medications (25, 26, 28–30). Further, CA has been reported to be associated with renal and electrolyte abnormalities in indomethacin-treated preterm infants (31). Fetal lambs following IA LPS administration have reduced nephron numbers (32, 33), implying possible sub-optimal renal functions in preterm infants born after CA (32, 34).

Preterm pig delivered at 90% gestation has been used as a clinically relevant model for preterm infants to investigate the effects of nutritional, microbial and immuno-modulatory interventions on organ functions and development (35, 36). Similar to preterm infants, preterm pigs possess multiple immaturities (e.g., underdeveloped gut, lungs, brain, and immune and cardiovascular systems) that make them highly susceptible to systemic infection and organ dysfunctions (37). Relative to rodents, the immune system in pigs is more similar to that in humans (38), with more human-like inflammatory responses to immune challenge (39). We have previously established a preterm pig model of CA to study effects of IA LPS on gut and lung injuries (35). In the current study, we hypothesized that IA LPS would also affect more distant organs like the kidneys, both at birth and during the neonatal period after preterm birth. We investigated clinical parameters, plasma markers of inflammation by proteomics, and kidney tissue evaluation of targets related to inflammation and innate and adaptive immune activation.

## Materials and Methods

### Animal Procedures

All animal procedures were approved by the Danish National Committee of Animal Experimentation (license number 2014-15-0201-00418). The animal experiment was conducted at the pig neonatal intensive care unit, the section for comparative pediatrics and nutrition, Copenhagen, Denmark. Three pregnant sows (Large White × Danish Landrace × Duroc) were operated (35) at day 103 of gestation (term at day 117 ± 2), and each fetus received an IA dose of 1 mg LPS/fetus (LPS group, *n* = 28, from *Escherichia coli* 055:B5, Sigma-Aldrich, Copenhagen, Denmark) in the area close to their mouth, or a control treatment (saline injection or no injection, CON group, *n* = 26). 3 days later, preterm piglets were delivered by cesarean section (90% of gestational age). The piglets were randomized according to their sex and birth weight into two subgroups within each treatment group. For each treatment, a fraction of piglets were euthanized right after delivery (*n* = 14 CON, 16 LPS), and the remaining pigs were reared by formula feeding until euthanasia at postnatal day 5 (*n* = 12 for both CON and LPS groups), as previously described (35). The preterm pig study was designed with parenteral nutrition and enteral nutrition using an infant formula to induce multiple clinically relevant complications, including NEC, shortly after birth (35). The study period of 5 postnatal days was selected to investigate the postnatal effect of IA LPS when the animals were not severely affected by formula-inducing complications. At euthanasia, the pig was initially anesthetized with an intramuscular injection of Zoletil mix (dose 0.1 ml/kg, including 0.25 mg/kg Zoletil, 0.25 mg/kg Butorphanol, 1.25 mg/kg Xylazin, and 1.25 mg/kg Ketamine), followed by an intracardiac injection of 20% Pentobarbital (Sigma-Aldrich, Copenhagen, Denmark) (35). Serum and urine samples at euthanasia (day 1 and day 5) were used for biochemistry (Advia 1800 Chemistry System. Siemens, Erlangen, Germany), and EDTA-treated plasma was used for proteomics. Kidney and liver tissues were snap-frozen and stored at −80°C for future analyses, and chorioamnion samples were fixed in paraformaldehyde 4% for histology. To balance the effect of litter and sex for treatment comparisons, one pig of each sex from each litter were selected for each treatment group in the validation stage. A random number selection method was used to choose the sample when more than one pig was eligible for each litter, sex and treatment, resulting in nine pigs in each group selected for the further validation analysis. Due to the sample availability, sample numbers may vary according to different sample types.

### Proteomic Analysis

The plasma samples were centrifuged at 1,000 × *g* for 5 min and measured in duplicates for protein content using a NanoDrop 2000 Spectrophotometer (Thermo Scientific, United States). The preparation procedures were based on a PVDF membrane-based proteomic sample processing method (MStern blotting) (40). Plasma protein (10 μg) was diluted into saturated urea followed by a reduction by 10 mM TCEP and alkylation by 50 mM chloroacetamide. Immobilon-P PVDF membrane (Millipore; 0.5 mm^2^) was incubated with saturated urea for 5 min and transferred to a 96-well, 1.5-ml deep well plate. Samples were incubated with the membrane for 10 min at ambient temperature using a thermomixer for continuous shaking. After incubation, samples were discarded, and the membranes washed with 150 μl, 50-mM ammonium bicarbonate (Ambic). Digestion buffer (100 μl, 5% TFE (v/v), 5% ACN (v/v), Trypsin 1:35 in 50 mM Ambic) was added to each sample and incubated overnight at 37°C in air incubator keeping high humidity. Tryptic peptides were recovered to collection tube followed by extraction of remaining peptides with 150 μl of 40% acetonitrile (ACN, v/v) 0.1% formic acid (FA; v/v). The pooled extracts were dried by vacuum centrifuge and resuspended with loading solvent (2% ACN, 0.1% trifluoroacetic acid, 0.1% FA in Milli-Q water) before loading into the LC-MS system.

The individual serum samples were randomized and sequenced on a hybrid trapped ion mobility spectrometry (TIMS) quadrupole time of flight (QToF) mass spectrometer, i.e., timsTOF in tims-off mode, (Bruker Daltonics, Bremen, Germany) coupled to modified nano-electrospray ion source (CaptiveSpray, Bruker Daltonics) with an applied voltage of 1800 V. Liquid chromatography was performed using a Dionex RSCL Proflow UHPLC (Dionex, Thermo Scientific, Waltham, United States) setup. Each sample was loaded onto a 2-cm reverse-phase C18-material trapping column and separated on a 75 cm analytical column (both from Acclaim PepMap100, Thermo Scientific). The liquid phase consisted of 96% solvent A (0.1% FA) and 4% solvent B (0.1% FA in ACN), at a flow rate of 300 nl/min. The peptides were eluted from the column by increasing to 8% solvent B and subsequently to 30% solvent B on a 35 min ramp gradient and introduced into the mass spectrometer by a Captivespray emitter for electrospray ionization (Bruker; Germany). The mass spectrometer was operated in positive mode with data-dependent acquisition (DDA), alternating between survey spectra and isolation/fragmentation spectra, using the Top20 method. All samples were analyzed in duplicates in a random order.

### Tissue Histology

Fixed chorioamnions were embedded in paraffin, sectioned, and stained with hematoxylin and eosin, as previously described (35). Frozen kidney tissues were sectioned and stained for myeloperoxidase (MPO), using rabbit anti-human MPO polyclonal antibody (AO398, Dako, Glostrup, Denmark), followed by anti-rabbit biotin-conjugated secondary antibody (Dako Denmark) and visualized with nickel-DAB. Frozen kidney sections were also stained for CD3, using CD3e-UNLB porcine primary antibody (monoclonal antibody PPT3) followed by Primary Antibody Enhancer, HRP Polymer (All from SouthernBiotech, Birmingham, United Kingdom), and visualized with nickel-DAB (Sigma-Aldrich) (41). Hematoxylin was used as counter-staining. All pictures were captured using Leica MC190 HD and positively stained area fraction in the total tissue area was quantified using ImageJ software (LOCI, University of Wisconsin).

### Gene Expressions by qPCR

Transcription of selected genes related to inflammation and innate and adaptive immune pathways in the liver and kidney tissues were determined by real-time RT-qPCR, using predesigned primers (Supplementary Table 4). Briefly, total RNA in tissue homogenates was isolated with RNeasy Lipid Tissue Mini Kit (Qiagen, Copenhagen, Denmark). RT-qPCR in kidney tissues was performed using QuantiTect SYBR Green PCR Kit (Qiagen) on a LightCycler 480 (Roche, Hvidovre, Denmark), and relative levels of target genes were normalized to the housekeeping gene HPRT1 (42). Gene expression in liver tissues was analyzed by 96.96 Dynamic Array Integrated Fluidic Circuits (Fluidigm, CA, United States), and relative expressions of target genes were normalized to the most stable reference genes (*GAPDH, HPRT, RPL13A, PPIA, TBP, B2M*, and *TBP1*), as previously described (35).

### Complement Proteins and Targets Related to Reactive Oxygen Species

The frozen kidney samples (Day 1: *n* = 9, Day 5: *n* = 8 in control and 9 in LPS) were also homogenized for analyses of superoxide dismutase (SOD) activity (SOD determination kit, Sigma-Aldrich, MO, United States), membrane attacking complex (MAC, C5b-C9, human C5b-9 ELISA kit, BD Biosciences Pharmingen, CA, United States), and peroxidation product malondialdehyde (MDA, MDA assay kit, Sigma-Aldrich).

### Western Blot

Renal protein expression level of leucine-rich alpha 2 glycoprotein 1 (LRG1) was analyzed by Western blot, using rabbit anti-human LRG1 polyclonal antibody antibodies (Sigma Aldrich, St. Louis, United States). Total proteins (Day1: *n* = 9, Day5: *n* = 8 in control and 9 in LPS) were extracted and separated by 10% Tricine SDS Gels (30 μg) and then transferred onto PVDF membrane (Millipore, Bedford, MA, United States). After blocking in 5% BSA 1 h at room temperature, the membranes were incubated with primary antibodies (1:1000 dilution) overnight at 4°C. The membranes were then washed twice with PBST, incubated with HRP-conjugated secondary antibody at 1:20000 dilutions for 1 h at room temperature, and visualized by the SuperSignal West Pico PLUS chemiluminescent substrate (Thermo Scientific^TM^, MA, United States). Mouse anti-porcine GAPDH monoclonal antibody (1:1000, Santa Cruz, CA, United States) was used as a loading control to generate the relative expression level of target protein. The Western blot results were quantified by Image J software (43). The target protein LRG1 level was analyzed by relative quantification and presented as a fold change to the loading control GAPDH.

### Data Analysis and Statistics

Mass spectrometry raw files were processed with MaxQuant (v 1.6.2.3) using the Andromeda search engine. Default MaxQuant settings were used using protein oxidation and N-terminal acetylation as variable modifications and carbamidomethyl cysteine as a fixed modification. Label-free quantification was performed using the MaxQuant label-free-quantification (MaxLFQ) algorithm.

Univariate analysis was applied to raw proteomics data (Supplementary Data Sheet 1) at each sampling time point using R studio 3.4.1 (R Studio, Boston, MA, United States). Briefly, a linear mixed effect model was fitted to each protein with treatment as the fixed factor, and litter as a random factor, using the lme4 package (44). To control the type *I* error, *p* value tests were further adjusted by false discovery rate (FDR, α = 0.2) into *q* values (45). Proteins with a *q* value ≤ 0.10 in any comparisons were chosen for the functional assignment. The protein interaction network analysis of biological processes and signaling pathways were performed using Cytoscape and ClueGO with a *p*-value cut-off *q* ≤ 0.1. Dunn’s Kruskal–Wallis multiple comparisons test of clinical data and Pearson’s correlation test were conducted in R. Results from qPCR, Western blot, and ELISA assays were analyzed by a linear mixed model, as described above, and a *p*-value < 0.05 was regarded as statistically significant. Data were presented as mean ± SEM.

## Results

### Effects of IA LPS on Amniotic Fluid, Fetal Membranes, Plasma Proteome, and Systemic Endpoints

**FIGURE 1:**
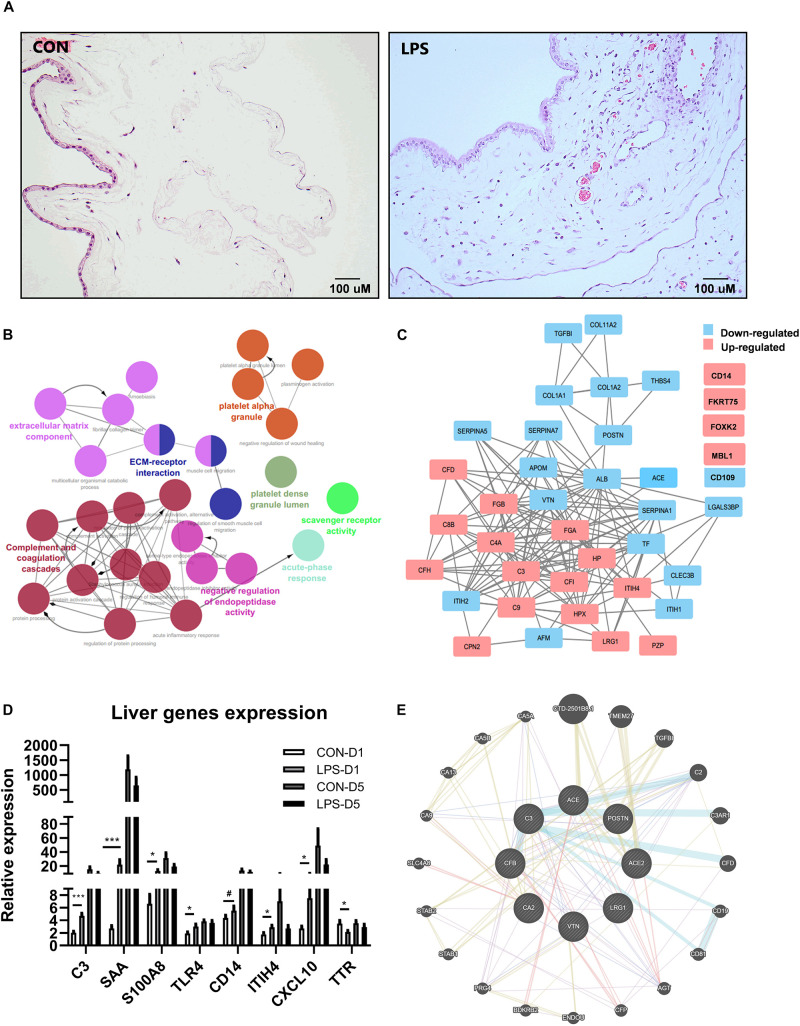
Effects of IA LPS exposure on chorioamnionitis (CA), changes of plasma proteome, and liver acute gene responses in preterm pigs at birth. **(A)** Inflammatory cell infiltration in the chorioamnion. **(B)** Pathway enrichment of differentially regulated proteins in plasma between LPS vs. CON pigs at birth (day 1). Every pathway is labeled according to the color. **(C)** Differentially regulated protein networks in plasma in LPS vs. CON pigs at birth (list of differential expressed proteins in Supplementary Table 2). **(D)** Changes of acute response genes in the liver of LPS pigs at birth. *n* = 10 in both groups. **(E)** A subgroup of differentially regulated proteins in plasma showed their functions related to kidney injury. The pathway enrichment was performed by GeneMANIA (84). Color code for **(E)** Blue: pathway interaction; Yellow: shared protein domains; Purple: co-expression; and Red: physical interactions. Width of the edge shows a weighted interaction network where each pair of genes is assigned an association weight (84). Data are presented as mean ± SEM. *, ***p < 0.05 and 0.001, respectively. ^#^*p* < 0.1.

Intra-amniotic LPS increased the number of infiltrated inflammatory cells in the chorioamnion (Figure 1A), similar to our previous report (35). Consistent with our previous report (35), amniotic fluid leukocyte counts and cytokines IL-6, IL-1β, TNF-α, and IL-10 were all highly elevated after IA LPS exposure (all *p* < 0.05, Supplementary Table 1).

Mass spectrometry-based plasma proteomics identified and annotated 245 proteins. IA LPS altered levels of 45 proteins at birth and two proteins at postnatal day 5. Analyses of pathway enrichment and protein-to-protein interaction revealed (Figures 1B,C) key regulated pathways, including complement and coagulation cascades, platelet activation, and acute phase response, indicating increased acute systemic inflammation in LPS pigs at birth. According to physiological functions, differentially regulated plasma proteins at birth were categorized into five groups: coagulation, acute phase response, protein processing, metabolism, and other functions (Supplementary Table 2). Acute-phase response to IA LPS at birth, but not on postnatal day 5, was confirmed by the increased expressions of liver genes. 64 genes in total were validated in transcription level in liver tissues (Supplementary Table 6), and eight genes were significantly changed in at birth, including *C3*, *SAA*, *S100A8*, *TRL4*, ITUH4, CXCL10, TTR, and *CD14* (Figure 1D); only two genes were significantly changed at postnatal day 5 (Supplementary Table 6). Notably, apart from the classical systemic inflammation-related proteins, a group of plasma proteins related to renal functions, including angiotensin-converting enzyme (ACE), carbonic anhydrase 2 (CA2), and LRG1 (Figure 1E), were also altered by IA LPS at birth.

### Markers of IA-LPS Induced Kidney Inflammation in Plasma, Urine, and Kidney Tissues

**FIGURE 2:**
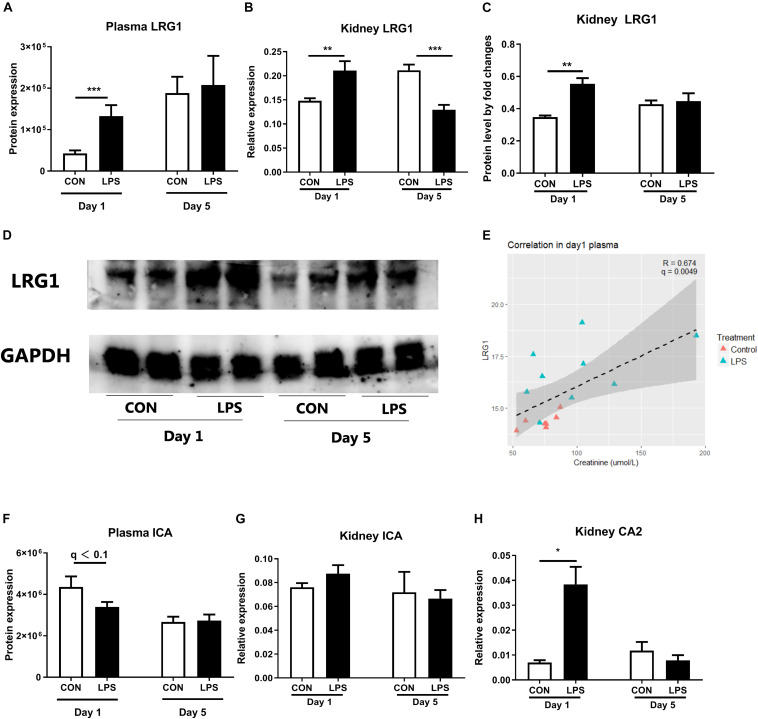
Markers of neonatal acute kidney injury. **(A)** The protein levels of LRG1 in plasma. **(B)** mRNA level of *LRG1* in kidney tissues. **(C,D)** The protein levels of LRG1 in kidney tissues by Western blot and the relative quantification to loading control GAPDH. (Day 1: *n* = 9, Day 5: *n* = 8 in control and 9 in LPS; samples were randomly selected to balance the effect of litter and sex for treatment comparisons; **E)** Correlation of plasma LRG1 levels and plasma creatinine levels. Samples with creatinine levels below detection range were not shown in the figure. Plasma LRG1 is positively correlated with levels of plasma creatinine levels. **(F)** The protein levels of ICA in plasma. **(G,H)** mRNA levels of *ICA* and *CA2* in kidney tissue. Data are presented as mean ± SEM. The Western blot results were quantified by Image J software (43). The target protein LRG1 level was analyzed by relative quantification and presented as a fold change to the loading control GAPDH. *q or *p* < 0.05; **q or *p* < 0.01, and ***q or *p* < 0.001. CA2, LRG1, and ICA, carbonic anhydrase 2, leucine-rich alpha-2-glycoprotein, and inhibitor of carbonic anhydrase.

As plasma proteomics showed IA LPS-induced changes of markers related to kidney inflammation, including LRG1 and inhibitor of carbonic anhydrase (ICA), these proteins and related targets were further examined in kidney tissues. Plasma LRG1 and renal *LRG1* expression after IA LPS exposure were both elevated at birth (*q* < 0.001 and *p* < 0.01, respectively, Figures 2A,B). This result was confirmed by Western blot analysis of LRG1 in kidney tissues (*p* < 0.05, Figures 2C,D). At postnatal day 5, the transcription level of LRG1 was significantly decreased in LPS pigs, but no changes were detected at protein level in both plasma and kidney tissues, probably reflecting differences in the post-transcriptional regulation of this gene between the two groups after birth.

Plasma LRG1 levels were also positively correlated with levels of urine Na + and plasma creatinine, a diagnostic marker of AKI (*r* = 0.6 and 0.647, respectively, *p* < 0.001, Figure 2E and Supplementary Figure 1). Despite lower levels of plasma ICA in LPS vs. CON pigs at birth, renal *ICA* mRNA levels were not altered by fetal LPS exposure (Figures 2F,G). However, the expression of *CA2* (carbonic anhydrase 2) in kidney tissues were elevated in LPS pigs at birth (*p* < 0.05, Figure 2H). Carbonic anhydrase is abundantly distributed in renal tissues and thought to play a pivotal role to catalyze the hydration–dehydration reaction of CO_2_ and bicarbonate reabsorption (46, 47). These findings suggest an IA LPS-induced imbalance in renal ICA and CA activity, potentially contributing to kidney inflammation.

**FIGURE 3:**
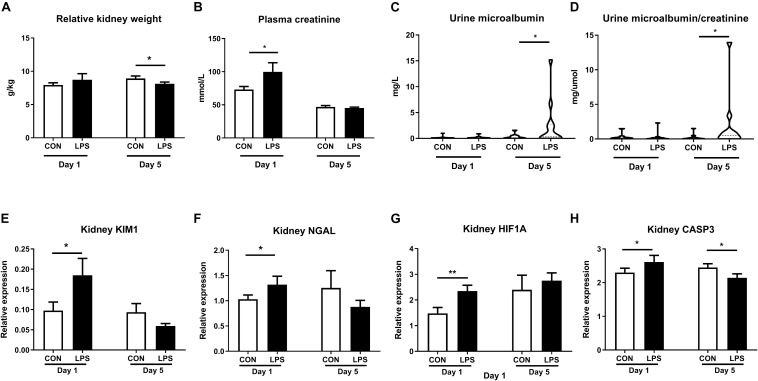
Effects of IA LPS on biochemical parameters and renal injury, hypoxia, and apoptosis markers. **(A)** Relative kidney weight, **(B)** Plasma creatinine, **(C)** Urinary microalbumin, **(D)** Urinary microalbumin/creatinine. **(E,F)** Renal expressions of two biomarkers for acute kidney injury *KIM1* and *NGLA*. **(G,H)** Renal expressions of hypoxia and apoptosis markers *HIF1A* and *CASP3*. Data are presented as mean ± SEM. *p < 0.05 and **p < 0.01. Serum samples: Day 1: *n* = 8 in control and 10 in LPS, Day 5: *n* = 13 in control and 15 in LPS; Urine samples: Day 1: *n* = 11, Day 5: *n* = 10 in control and 12 in LPS. KIM1, NGLA, HIF1A, and CASP3: kidney injury molecule-1, neutrophil gelatinase-associated lipocalin, hypoxia-inducible factor 1- alpha, and caspase 3.

Intra-amniotic LPS also altered clinical and biochemical parameters and other kidney injury markers both at birth and postnatal day 5. Relative kidney weight showed no difference at birth but decreased in LPS vs. CON pigs at postnatal day 5 (*p* < 0.05, Figure 3A). Plasma creatinine was elevated by LPS exposure only at birth (*p* < 0.05) but not on day 5 (Figure 3B). Both the microalbumin levels and the ratio of microalbumin over creatinine in urine were higher on day 5 in LPS vs. CON pigs (*p* < 0.05, Figures 3C,D). The renal pathological score and glomerular sizes were unavailable due to a lack of high-quality formalin fixation tissue in the current study. Other markers of kidney injury (48, 49) (kidney injury molecule-1, *KIM-1*, and neutrophil gelatinase-associated lipocalin, *NGLA*), hypoxia, and apoptosis (*HIF1A* and *CASP3*) were also evaluated with consistently higher levels in LPS vs. CON pigs at birth (*p* < 0.05, Figures 3E–H). These biochemical and qPCR data further confirmed renal inflammation in LPS pigs at birth, while differences in kidney weight and urine microalbumin potentially indicate renal IA LPS effects persisting until postnatal day 5.

### IA LPS Stimulates Renal Innate Immune Activation

**FIGURE 4:**
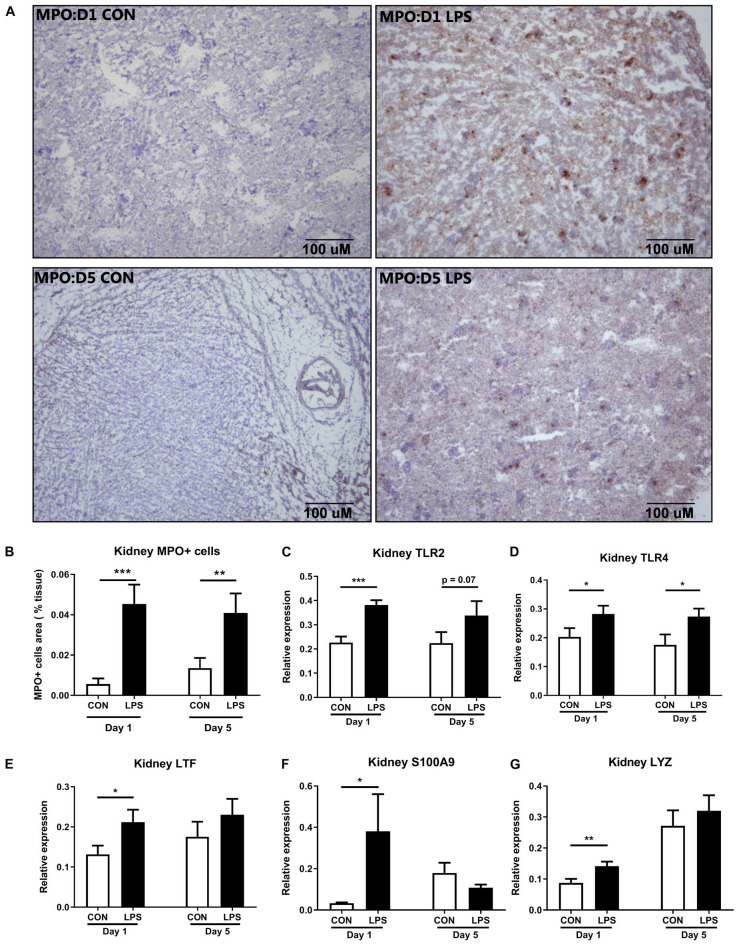
Kidney innate immune response at birth and postnatal day 5 following IA LPS exposure. **(A,B)** MPO^+^ stained cells (brown) in the frozen kidney, **(C–G)** mRNA levels of toll-like receptors (*TLR2* and *TLR4*) and neutrophil/macrophage components (*LTF*, *S100A9*, and *LYZ*) in kidney tissues. Data are presented as mean ± SEM. *p < 0.05 and **p < 0.01. Day 1: both *n* = 9, Day 5: *n* = 8 in control and 9 in LPS. The scale bars in **(A)** represent 100 μm. LTF, lactoferrin; TLR, toll-like receptor; and LYZ, lysozyme.

Next, we sought to examine innate immune responses in kidney tissues modulated by IA LPS. The area of infiltrated MPO^+^ cells (a marker of macrophages and/or neutrophils) was highly elevated both at birth and postnatal day 5 in LPS vs. CON pigs (*p* < 0.001 and 0.01, respectively, Figures 4A,B). The same trend was observed for the expression levels of *TLR2* and *TLR4* (all *p* < 0.05 except 0.07 for *TLR2* on day 5, Figures 4C,D). mRNA levels of the neutrophil and/or macrophage components *LTF* (product of the secondary granules of neutrophils), *S100A9* (an inflammation marker released by neutrophils and macrophages), and *LYZ* (a marker presented in all three types of human neutrophil granules) were also higher in LPS vs. CON pigs at birth (all *p* < 0.05, Figures 4E–G). These data indicate that IA LPS stimulated innate inflammatory responses in kidney tissues both at birth and postnatal day 5 via infiltration of neutrophils and/or macrophages.

### IA LPS Activates the Fetal and Postnatal Renal Complement System

**FIGURE 5:**
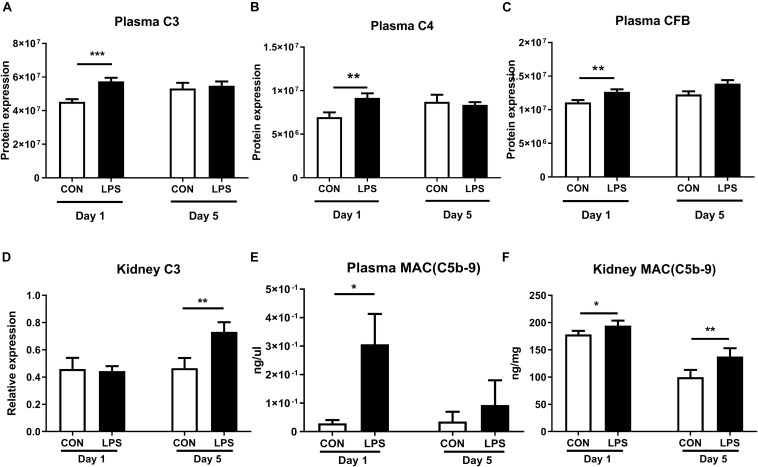
Kidney complement activation induced by IA LPS. **(A–D)** Protein levels of C3, C4, CFB in plasma, and mRNA levels of *C3* in kidneys. **(E,F)** Membrane attacking complex (MAC and C5b-9) in plasma and kidneys. Data are presented as mean ± SEM. *q or *p* < 0.05; **q or *p* < 0.01, and ***q or *p* < 0.001.

As IA LPS-induced kidney effects were strongly associated with innate immune activation, we next assessed the status of the renal complement system, a crucial component of the innate immunity. Plasma proteomics data at birth revealed increased levels of C3, C4, and CFB in LPS vs. CON pigs (all *q* < 0.01, Figures 5A–C). In kidney tissues, the mRNA levels of *C3* were elevated in LPS vs. CON pigs on day 5 (*p* < 0.01, Figure 5D). To verify the actions of the complement system, the final complement product, membrane attacking complex (MAC) C5b-C9 was determined with higher levels in both plasma and kidneys at birth and also higher levels in the kidneys at postnatal day 5 in LPS vs. CON pigs (all *p* < 0.05, Figures 5E,F).

### IA LPS Alters the Renal ACE System and Increases Renal Oxidative Stress

**FIGURE 6:**
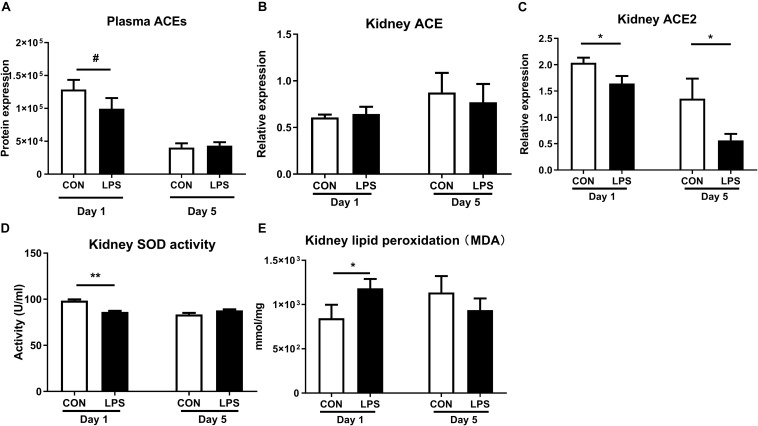
Effects of IA LPS on the renal renin-angiotensin system and generation of reactive oxygen species. **(A)** Protein levels of ACEs in plasma. **(B,C)** mRNA levels of *ACE* and *ACE2* in kidney tissues. **(D)** SOD activity in kidney tissue. **(E)** Lipid peroxidation (malondialdehyde, MDA) level in kidney tissue. Data are presented as mean ± SEM. *q or *p* < 0.05 and **q or *p* < 0.01. ACE, angiotensin-converting enzyme; SOD, superoxide dismutase.

As plasma proteomics revealed lower levels of ACEs in LPS vs. CON pigs at birth (*q* < 0.1, Figure 6A), we also sought to determine IA LPS effects on status of the renal ACE system and its role in the production of oxidative stress. ACEs are key components in the renin-angiotensin system (RAS) that modulates blood pressure, reactive oxygen species (ROS), and renal status (50). Especially, angiotensin II is damaging to renal tubules via ROS generation and inflammation and may contribute to glomerular injury and proteinuria (51, 52), while ACE2 functions to degrade angiotensin II, thereby decreasing ROS generation (50). Our data showed no IA LPS effects on *ACE* levels but *ACE2* levels were lower in the kidneys of LPS vs. CON pigs, both at birth and postnatal day 5 (*p* < 0.05, Figures 6B,C). LPS pigs at birth also showed increased oxidative stress with decreased renal levels of SOD activity and increased renal levels of MDA, relative to CON pigs (*p* < 0.01 and 0.05, Figures 6D,E). These data suggest that IA LPS elevated ROS activity in kidney tissues with potential involvement of the renal ACE system.

### IA LPS Stimulates Renal Adaptive Immune Activation

**FIGURE 7:**
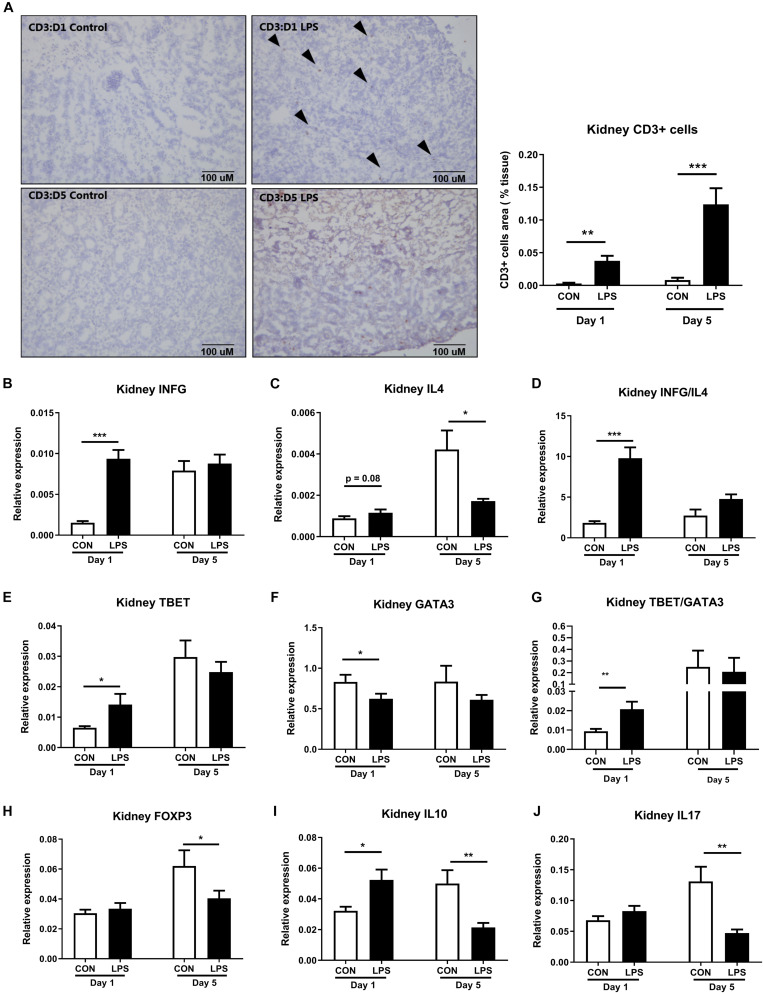
Kidney adaptive immune responses both at birth and postnatal day 5 following IA LPS exposure. **(A)** The density of renal CD3^+^ cells (T cells). **(B–J)** mRNA levels of Th1-polarizing cytokine *IFNG*, Th2-polarizing cytokines *IL4* and *IL10*, the ratio of Th1/Th2 cytokines *IFNG*/*IL4*, Th1 transcription factor *TBET*, Th2 transcription factor *GATA3*, the ratio of Th1/Th2 transcription factors *TBET*/*GATA3*, markers of Treg *FOXP3*, and marker of Th17 *IL17*. Data are presented as mean ± SEM. *p < 0.05; **p < 0.01, and ***p < 0.001. The scale bars in **(A)** represent 100 μm.

Likewise, the renal adaptive immune responses were also modulated by IA LPS exposure. The density of renal CD3^+^ cells (T cells) was significantly up-regulated in LPS pigs both at birth and postnatal day 5 (*P* < 0.01 and 0.001, Figure 7A). At birth, the kidneys of LPS pigs showed increased Th1 responses, with higher mRNA levels of Th1-polarizing cytokines, ratio of Th1/Th2 cytokines (*IFNG*, ratio of *IFNG*/*IL4*), Th1 transcription factor (*TBET*), and ratio of Th1/Th2 transcription factors (*TBET/GATA3*), as well as decreased mRNA levels of Th2 transcription factor (*GATA3*, all *p* < 0.05, Figures 7B–G). At postnatal day 5, LPS pigs had lower renal mRNA levels of Th2-polarizing cytokines (*IL4, IL10*) and marker of regulatory T cells (*FOXP3* expression, all *p* < 0.05, Figures 7C,H,I). *IL17* expression was also reduced in LPS vs. CON pigs on day 5 (*p* < 0.05, Figure 7J). These data indicate increased fetal and postnatal renal inflammation in LPS vs. CON pigs with the activation of both innate and Th1-mediated immunity. In contrast to the systemic acute phase response (as assessed by plasma proteomics and liver gene expression above), the renal effects of IA LPS exposure persisted in the postnatal period, at least until day 5.

## Discussion

Preterm infants born with CA are often at increased risk of developing neonatal morbidities and organ injuries, including renal inflammation and AKI (21% of hospitalized neonates) (53). Due to the disruption of natural fetal nephrogenesis at a crucial developmental time point, the kidney size and nephron number are reduced in surviving premature infants (54). Fetal insults, including CA that is often accompanied with preterm birth, elicit inflammation in multiple organs and may further worsen the renal outcomes. Early management of kidney inflammation may ameliorate growth failure and suboptimal neurodevelopmental outcomes for preterm infants (55, 56), but reliable diagnostic markers for neonatal kidney diseases are lacking, and underlying mechanisms of the diseases are poorly understood. In the current study with preterm pigs, we demonstrated for the first time that CA, induced by IA LPS exposure, resulted in renal inflammation both at birth and 5 days after preterm birth with the involvement of innate and adaptive immune activation. Our data imply that prenatal insults may play a critical role in determining neonatal kidney outcomes and may explain the high incidence of AKI in preterm infants (46, 57–60), although reduced gestational age at birth is also a risk factor (61).

**FIGURE 8:**
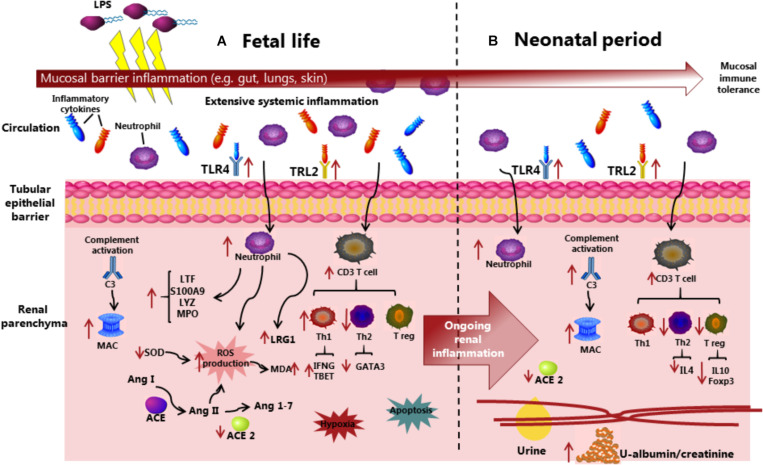
Overview of the suggested mechanisms that IA LPS induces fetal and neonatal acute kidney injury before and after preterm birth. Compared with control pigs, LPS preterm pigs showed compromised kidney function, increased immune cell infiltration with complement system activation and production of reactive oxygen species, and increased level of hypoxia and apoptosis in the kidneys. The inflammatory state in kidney tissues is persistent from fetal to neonatal periods, in contrast with the acute mucosal inflammatory response in the gut and lungs being attenuated at postnatal day 5. ACE, Angiotensin-converting enzyme; Ang, Angiotensin; C3, Complement protein 3; LPS, Lipopolysaccharides; LRG1, Leucine-rich alpha-2-glycoprotein 1; MAC, Membrane attack complex; MDA, Malondialdehyde; MPO, Myeloperoxidase; ROS, Reactive oxygen species; SOD, Superoxidase dismutase; Th, T helper cell; Treg, Regulatory T cell; and TLR, Toll-like receptor.

First, we demonstrated that prenatal IA LPS induced CA-like responses in the fetal membranes, marked systemic immune responses at birth, and altered plasma levels of markers related to kidney injuries, including LRG1, ACE, and ICA. Via evaluation of biochemical parameters, together with analyses of endpoints related to inflammatory pathways in kidney tissues, we showed that IA LPS exposure resulted in renal inflammation not only at birth but also in the neonatal period after preterm birth. Considering that IA LPS-induced lung and gut inflammation mainly occurs at birth (35), our data indicate that the preterm kidneys are relatively sensitive to prenatal endotoxin exposure. This is noteworthy also from the perspective that mucosal surfaces in the gut, lungs, and skin have more direct contact with endotoxin and inflammatory molecules in the amniotic sac following IA LPS or IA inflammation. Conversely, IA inflammation may affect systemic organs, such as kidneys, more gradually and for a longer time, following the translocation of inflammatory signals across the immature gut, lung, and skin barriers. Based on our results, Figure 8 was suggested to illustrate possible mechanisms whereby prenatal insults may lead to fetal and neonatal kidney inflammation in preterm neonates.

At birth, IA LPS-exposed preterm pigs had elevated levels of plasma creatinine, a commonly used diagnostic marker for AKI (62) and also increased renal transcription levels of two well-known kidney injury markers (*KIM1* and *NGAL*) (63–65). The renal injury in these pigs seemed to persist to postnatal day 5 when urinary levels of microalbumin and the ratio of microalbumin/creatinine were elevated, together with decreased relative kidney weight. The mechanisms may include strong infiltrations of macrophages and/or neutrophils (MPO^+^ cells) with elevated expression levels of *TRL2* and *TRL4* in kidney tissues both at birth and postnatal day 5. This was further supported by elevated expression levels of the neutrophil and/or macrophage components *LTF*, *S100A9* and *LYZ* in LPS pigs at birth. As prenatal inflammation is often associated with hypoxia-ischemia (HI) and apoptosis in internal organs (66), we also validated the corresponding markers and found elevated expressions of *HIF1A* and *CASP3* in the kidneys of LPS vs. CON pigs at birth.

Mucosal surfaces, like skin, gut, and lungs, may adapt more rapidly to fetal endotoxin exposure than the kidneys, consistent with the need of epithelia to tolerate the abrupt changes from a relatively sterile *in utero* environment to a microbes-rich environment *ex utero*. In the kidneys, an IA LPS-induced innate inflammatory response via neutrophil/macrophage activation was further potentiated by Th1 adaptive immune responses, i.e., dramatically elevated levels of infiltrated CD3^+^ T cells, expression of *IFNG* (Th1 cytokine), *TBET* (Th1 transcription factor), and ratios of *IFNG/IL4* and *TBET/GATA3*, together with decreased expression levels of *GATA3* (Th2 transcription factor) at birth. Although these Th1 markers were not different between LPS and CON pigs on day 5, LPS pigs showed lower expression levels of anti-inflammatory genes, including *IL4, IL10* (Th2 cytokines), and *FOXP3* (regulatory T cell transcription factor). Likewise, the reduced *FOXP3* and *IL10* levels further demonstrate reduced regulatory T cell population in postnatal day 5. Thus, the adaptive immune responses may also last at least until day 5, probably via the action of other Th1 cytokines or Th1-enhanced activities of neutrophils/macrophages.

On the other hand, RAS, regulated by ACE molecules, may also be involved in IA LPS-induced renal inflammation as plasma ACEs were down-regulated at birth in LPS pigs and the renal mRNA level of *ACE2* was down-regulated at both time points. The balance between ACE and ACE2 controls the production of angiotensin II (ANG II) and angiotensin-(1–7) (ANG-(1–7) (67). Decreased ACE2 level has been reported to cause ANG II accumulation, thereby enhancing ROS and oxidative damage. Consistent with this, we showed IA LPS-induced reduction of SOD enzyme activity (enzyme neutralizing ROS), as well as increased levels of malondialdehyde [MDA, a marker of lipid peroxidation leading to tissue damage (68)] in the kidneys. In LPS pigs, the neutrophil/macrophage and complement activation in kidney tissues may generate vast amounts of ROS while the renal ACE and anti-oxidative systems could be less efficient, potentially leading to increased ROS-induced tissue damage.

The current study results also suggested plasma markers and therapeutic targets of prenatal inflammation and related organ responses including renal inflammation, namely, LRG1 and ICA (increased and decreased by IA LPS, respectively). LRG1 is a neutrophil component and has been reported to play a crucial role in immune responses, cell proliferation and apoptosis, neovascularization, and hypoxia (69–73). LRG1 has been shown to be elevated in the urine of patients with IgA nephropathy and chronic kidney disease (74, 75). In our study, both mRNA and protein levels of LRG1 were increased in the kidneys of LPS pigs at birth and correlated with plasma creatinine, a classical marker of AKI. LRG1 may also play a crucial role in the progression of hypoxia by regulating HIF1A expression (76). As *HIF1A* was also up-regulated by IA LPS in the current study, we speculate that LRG1-mediated hypoxia via HIF-1α activation also contributes to kidney injury and that LRG1 may be a promising diagnostic and therapeutic target of prenatal inflammation and organ responses including neonatal renal inflammation. On the other hand, the ICA is a CA inhibiting protein and pH (acid-base balance) regulating enzyme in multiple cells and tissues (77–79), although in humans it may only be a pseudogene (80). Instead, we found elevated expression of renal carbonic anhydrase II (*CA2*) in LPS pigs at birth and speculated that the imbalance between ICA and CA2 may enhance bicarbonate generation and low tissue pH, contributing to inflammation-related kidney dysfunction.

The nephrogenesis in pig occurs during both intrauterine and extrauterine periods, from day 29 of postconceptional age to postnatal day 21 (81), whereas the nephrogenesis in human completes before gestational week 36. No new nephrons are formed after gestational week 36 over the lifetime in humans (82). We speculate that some postnatal parameters related to inflammation and oxidative stress that were reduced over time may be due to the ongoing extrauterine nephrogenesis in pigs or partly resolved inflammation following fetal acute responses.

In conclusion, prenatal IA LPS exposure induced clear signs of fetal and neonatal kidney inflammation in preterm pigs. In contrast to conditions in the lungs, gut, and liver, the kidney inflammatory effects persisted into the postnatal period, possibly driven by sustained activation of both innate and adaptive immune cells. Plasma LRG1 may be a new promising diagnostic and therapeutic target for prenatal inflammation and organ responses including neonatal renal inflammation. Preterm infants born after CA may suffer from dysfunctions of multiple organs, including kidneys, that require special care and treatments to prevent further short- and long-term complications.

## Data Availability Statement

The datasets presented in this study can be found in online repositories. The names of the repository/repositories and accession number(s) can be found in the article/Supplementary Material. The mass spectrometry proteomics data have been deposited to the ProteomeXchange Consortium via the PRIDE (83) partner repository with the dataset identifier PXD016013.

## Ethics Statement

The animal study was reviewed and approved by The Danish National Committee of Animal Experimentation.

## Author Contributions

TM conceived the study, analyzed the proteomic data, performed tissue analyses, and prepared the manuscript. P-PJ conceived the study, analyzed and interpreted the data, and prepared the manuscript. AS performed the proteomics analysis and protein annotation. PTS conceived the study, interpreted the data, and prepared the manuscript. KS performed the liver gene expression experiment and data analysis. DNN conceived the study, performed the animal experiment, interpreted the data, and prepared the manuscript. All authors drafted the work, revised it critically for important intellectual content, and approved the final version.

## Conflict of Interest

The authors declare that the research was conducted in the absence of any commercial or financial relationships that could be construed as a potential conflict of interest.

## Supplementary Material

Supplementary Figure 1 Correlations between plasma LGR1 levels and biochemical parameters.

Supplementary Table 1 Amniotic fluid cytokines and infiltrated inflammatory cells in the chorioamnion.

Supplementary Table 2 Differentially regulated proteins in plasma between LPS and CON pigs at birth (day 1).

Supplementary Table 3 Differentially regulated proteins in plasma between LPS and CON pigs at postnatal day 5.

Supplementary Table 4 Primer sequence of selected genes analyzed in kidney tissues.

Supplementary Table 5 Primer sequences for liver gene analysis.

Supplementary Table 6 Liver gene expressions.

## Abbreviations

ACE: Angiotensin-converting enzyme
ACE2: Angiotensin I Converting Enzyme 2
AKI: Acute kidney injury
C3: Complement C3
C3: Complement C3
C4: Complement C4
CA: Chorioamnionitis
CA: Carbonic anhydrase 2
CASP3: Caspase 3
CD14: CD14 molecule
CKD: Chronic kidney disease
FDR: False discovery rate
FOXP3: Forkhead box P3
GA: Gestational age
GATA3: transcription factors GATA3
HIF1A: Hypoxia inducible factor 1 subunit alpha
HPRT1: Hypoxanthine-guanine phosphoribosyltransferase
ICA: Carbonic anhydrase inhibitor
IHC: Immunohistochemistry
IL10: Interleukin 10
IL17: Interleukin 17
IL4: Interleukin 4
INFr: Interferon-gamma
KIM-1: Kidney injury molecule-1
LPS: Lipopolysaccharide
LRG1: Leucine-rich alpha-2-glycoprotein 1
LTF: Lactotransferrin
LYZ: Lysozyme
MDA: Malondialdehyde
MS: Mass spectrometry
NGLA: Neutrophil gelatinase-associated lipocalin
RAS: Renin-angiotensin system
RT-qPCR: Reverse transcription quantitative real-time PCR
ROS: Reactive oxygen species
S100A9: S100 Calcium Binding Protein A9
SEM: Standard error of the mean
SOD: Superoxide dismutase
SAA: Serum Amyloid A
TBET: Transcription factors T-bet
TLR: Toll-like receptor
TLR2: Toll-like receptor 2
TLR4: Toll-like receptor 4
TNF- α: Tumor necrosis factor- α.
