# Supplementary material for: Prenatal Endotoxin Exposure Induces Fetal and Neonatal Renal Inflammation via Innate and Th1 Immune Activation in Preterm Pigs
Source: Front Immunol. 2020 Sep 30;11:565484. doi: 10.3389/fimmu.2020.565484 (PMC7643587; doi:10.3389/fimmu.2020.565484)
Supplement: Supplementary Figure 1 — Correlations between plasma LGR1 levels and biochemical parameters. [file Table_1.DOCX]

**Supplemental information**

**Prenatal endotoxin exposure induces fetal and neonatal renal inflammation via innate and Th1 immune activation in preterm pigs**

Tik Muk, Ping-Ping Jiang, Allan Stensballe, Kerstin Skovgaard, Per Torp Sangild, Duc Ninh Nguyen*

* Correspondence: Duc Ninh Nguyen, Section for Comparative Pediatrics and Nutrition, Department of Veterinary and Animal Sciences, University of Copenhagen.

Email: dnn@sund.ku.dk

**Figure S1** Correlations of plasma LGR1 levels and biochemical parameters

**Figure S1** Correlations of plasma LGR1 levels and biochemical parameters

**A**

**
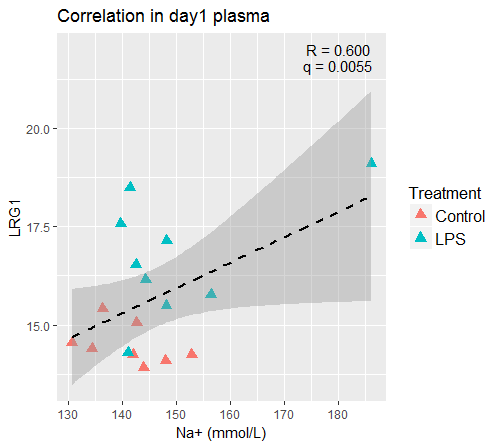
**

**Figure S1 (A)** Correlation of plasma LRG1 levels and urine Na^+^ levels. Plasma LRG1 is positively correlated with levels of urine Na^+^ levels.
