# Supplementary material for: Prenatal Endotoxin Exposure Induces Fetal and Neonatal Renal Inflammation via Innate and Th1 Immune Activation in Preterm Pigs
Source: Front Immunol. 2020 Sep 30;11:565484. doi: 10.3389/fimmu.2020.565484 (PMC7643587; doi:10.3389/fimmu.2020.565484)
Supplement: Supplementary Table 1 — Amniotic fluid cytokines and infiltrated inflammatory cells in the chorioamnion. [file Table_2.docx]

**Supplemental information**

**Prenatal endotoxin exposure induces fetal and neonatal renal inflammation via innate and Th1 immune activation in preterm pigs.**

Tik Muk, Ping-Ping Jiang, Allan Stensballe, Kerstin Skovgaard, Per Torp Sangild, Duc Ninh Nguyen*

* Correspondence: Duc Ninh Nguyen, Section for Comparative Pediatrics and Nutrition, Department of Veterinary and Animal Sciences, University of Copenhagen.

Email: dnn@sund.ku.dk

**Table S1** Amniotic fluid cytokines and infiltrated inflammatory cells in the chorioamnion

**Table S2** Differential regulated proteins in day 1 plasma

**Table S3** Differential regulated proteins in day 5 plasma

**Table S4** Primer sequence of selected genes analyzed in kidney tissues

**Table S5** Primer sequences for liver gene analysis

**Table S6** Liver gene expressions

**Table S1.** Clinical data of the piglets used in the proteomics analysis

|  | **Day 1** | | | **Day 5** | |  |
| --- | --- | --- | --- | --- | --- | --- |
|  | **Control** | | **LPS** | **Control** | **LPS** |  |
| **Chorioamnion** |  |  | |  |  | |
| Infiltrated inflammatory cells (Score) | 0 (0 - 1) | 2 (1 - 3) * | | - | - | |
| **Amniotic fluid** |  |  | |  |  | |
| Leukocyte counts (cells/mL) | 113 ± 9 | 1398 ± 34 *** | | - | - | |
| IL6 (pg/mL) | 119.58 ± 18.47^a^ | 728.95 ± 97.14 *** | | - | - | |
| TNFA (pg/mL) | 26.64 ± 2.93 | 478.92 ±103.66 *** | | - | - | |
| IL10 (pg/mL) | 4.23 ± 2.93 | 81.81 ± 12.24 ** | | - | - | |
| IL1β (pg/mL) | 65.53 ± 7.6 | 433.59 ± 135.47 *** | | - | - | |
| IL8 (pg/mL) | 205.53 ± 60.61 | 3155.68 ±549.8 *** | | - | - | |
| **Others** |  |  | |  |  | |
| NEC score | 3.1 ± 0.1^a^ | 1.4 ± 0.2^b^ | |  | 2.2 ± 0.2^c^ | |

Data presented as mean ± SEM or median (1^st^ - 3^rd^ quantile); data bearing same letter are not significantly different from each other. Scoring system for infiltrated inflammatory cells is: 0, no or few inflammatory cells (no chorioamnionitis); 1, significant amounts of inflammatory cells restricted to chorion (stage 1 of chorioamnionitis); 2, both chorion and amnion (stage 2); or 3, necrotizing amniotic epithelial cells (stage 3). Scoring system for NEC is: 1, normal appearance; 2, mild hyperemia and/or edema; 3, extensive hyperemia, edema, and local hemorrhage; 4, extensive hemorrhage and/or pneumatosis intestinalis; 5, hemorrhage with local necrosis; and 6, severe hemorrhage and necrosis. The score system of infiltrated inflammatory cells and NEC was presented previously [1]. Cytokines were analyzed by porcine-specific ELISA-Duo set kits (R&D Systems).

**Table S2** Differential plasma proteins in day 1

|  |  |  | **Abundance^1^** | |  | |  |
| --- | --- | --- | --- | --- | --- | --- | --- |
| **UniProt ID** | **Gene name** | **Protein name** | **Control** | **LPS** | ***q* value** | |  |
|  |  | **Acute immune response** |  |  |  | |  |
| F1S790_PIG | C8B | Complement C8 Beta Chain | 13.15±1.33 | 14.11±1.04 | 0.060 | |  |
| CO3_PIG | C3 | Complement C3 | 25.41±0.05 | 25.75±0.05 | 0.000 | |  |
| CFAB_PIG | BF;CFB | Complement factor B | 23.39±0.04 | 23.58±0.04 | 0.008 | |  |
| CFAD_PIG | CFD | Complement factor D | 16.94±0.66 | 17.01±0.22 | 0.131 | |  |
| HPT_PIG | HP | Haptoglobin; | 17.27±0.35 | 20.01±0.54 | 0.001 | |  |
| F1SMJ6_PIG | C9 | Complement C9 | 16.12±0.62 | 17.22±0.17 | 0.120 | |  |
| F1RQH9_PIG | CD109 | CD109 Molecule | 16.42±0.68 | 15.71±0.66 | 0.146 | |  |
| F1RQW2_PIG | C4 | Complement C4 | 22.62±0.11 | 23.07±0.09 | 0.002 | |  |
| F1S643_PIG | CFH | Complement factor H | 20.56±0.12 | 20.86±0.09 | 0.100 | |  |
| F1S133_PIG | CFI | Complement factor I | 20.67±0.08 | 20.94±0.07 | 0.060 | |  |
| A2SW51_PIG | CD14 | Monocyte differentiation antigen CD14 | 19.94±0.09 | 20.21±0.11 | 0.139 | |  |
| A0A287A1S6_PIG | COL1A1 | Collagen alpha-1(I) chain preproprotein | 19.92±0.22 | 18.83±0.23 | 0.002 | |  |
| F1SFA7_PIG | COL1A2 | Collagen alpha-2(II) chain preproprotein | 19.34±0.28 | 18.29±0.33 | 0.021 | |  |
| F1SH96_PIG | ITIH1 | Inter-alpha-trypsin inhibitor heavy chain H1 | 23.28±0.08 | 23±0.08 | 0.057 | |  |
| F1RUM4_PIG | ITIH2 | Inter-alpha-trypsin inhibitor heavy chain H2 | 23.9±0.07 | 23.71±0.07 | 0.109 | |  |
| F1RMN7_PIG | HPX | Hemopexin | 17.83±0.31 | 19.74±0.22 | 0.000 | |  |
| F1RXM6_PIG | SERPINA7 | Thyroxine-binding globulin | 19.62±0.17 | 18.99±0.2 | 0.060 | |  |
| F1S012_PIG | FOXK2 | Forkhead box protein K2 | 14.86±1.04 | 18.15±0.38 | 0.027 | |  |
| BGH3_PIG | TGFBI | Transforming growth factor-beta-induced protein ig-h3 | 16.83±0.7 | 16.69±0.25 | 0.112 | |  |
| VTNC_PIG | VTN | Vitronectin | 18.11±0.27 | 16.59±1.21 | 0.131 | |  |
| F1SCE3_PIG | SERPINA5 | Plasma serine protease inhibitor preproprotein | 19.86±0.11 | 19.39±0.11 | 0.026 | |  |
| F1RX37_PIG | FGB | Fibrinogen beta chain | 24.49±0.1 | 24.73±0.11 | 0.100 | |  |
| I3LJA6_PIG | FGA | Fibrinogen Alpha Chain | 18.88±0.18 | 19.43±0.16 | 0.086 | |  |
| F1S2C1_PIG | COL11A2 | Collagen Type XI Alpha 2 Chain | 18.47±0.26 | 18.01±0.25 | 0.141 | |  |
| A0A287A604_PIG | LGALS3BP | Galectin 3 Binding Protein | 17.93±0.14 | 17.61±0.72 | 0.062 | |  |
| ITIH4_PIG | ITIH4 | Inter-alpha-trypsin inhibitor heavy chain H4 | 25.85±0.08 | 26.11±0.06 | 0.060 | |  |
|  |  | **Coagulation** |  |  |  | |  |
| F1SK70_PIG | PROS1 | Vitamin K-dependent protein S isoform 2 preproprotein | 20.14±0.09 | 19.81±0.14 | 0.120 | |  |
|  |  | **Cellular processing** |  |  |  | |  |
| F1SEY1_PIG | LOC100518647 | Alpha-mannosidase | 15.15±0.82 | 15.36±0.62 | 0.073 | |  |
| F1RF27_PIG | THBS4 | Thrombospondin 4 | 20.78±0.15 | 20.39±0.15 | 0.126 | |  |
| F1SRC8_PIG | CLEC3B | Tetranectin | 20.93±0.11 | 20.57±0.09 | 0.060 | |  |
| A1AT_PIG | SERPINA1 | Alpha-1-antitrypsin | 24.86±0.1 | 24.56±0.11 | 0.120 | |  |
| I3LQ17_PIG | PZP | Pregnancy zone protein | 18.84±0.22 | 19.34±0.16 | 0.109 | |  |
|  |  | **Metabolism** |  |  |  | |  |
| APOM_PIG | APOM | Apolipoprotein M | 20.48±0.11 | 20.05±0.11 | 0.030 | |  |
| F1RUM1_PIG | AFM | Afamin | 21.42±0.08 | 21.08±0.08 | 0.026 | |  |
| I3LF89_PIG | CPN2 | Carboxypeptidase N Subunit 2 | 16.77±0.17 | 17.83±0.2 | 0.002 | |  |
| ICA_PIG | ICA | Inhibitor of carbonic anhydrase | 21.87±0.13 | 21.61±0.1 | | 0.113 | |
| F1RRW4_PIG | ACE | Angiotensin-converting enzyme | 13.25±1.34 | 13.73±1.19 | 0.094 | |  |
|  |  | **Others** |  |  |  | |  |
| F1S7K2_PIG | LRG1 | Leucine-rich alpha-2-glycoprotein 1 | 13.4±0.91 | 15.13±0.91 | 0.002 | |  |
| I3LDM1_PIG | POSTN | Periostin isoform 3 | 19.03±0.3 | 18.09±0.24 | 0.060 | |  |
| ALBU_PIG | ALB | Serum albumin | 26.53±0.1 | 26.29±0.12 | 0.147 | |  |
| F1RX36_PIG | LOC102158263 |  | 24.34±0.09 | 24.57±0.1 | 0.065 | |  |
| F1SCC9_PIG | LOC106504545 |  | 21.55±0.39 | 20.34±0.56 | 0.113 | |  |
| F1SCC7_PIG | LOC100156325 |  | 19.98±0.29 | 22.16±0.14 | 0.000 | |  |
| ND | LOC100153899 |  | 11.44±1 | 19.91±0.33 | 0.000 | |  |

**^1^**, Abundance is base-2 logarithm transformed. 2, ND, not detected.

**Table S3** Differential plasma proteins in day 5.

|  |  |  | **Abundance^1^** | |  |
| --- | --- | --- | --- | --- | --- |
| **UniProt ID** | **Gene name** | **Protein name** | **Control** | **LPS** | ***q* value** |
| F1SM72_PIG | NCAM1 | Neural Cell Adhesion Molecule 1 | 13.57±1.83 | 12.95±2.26 | 0.033 |
| F1SCD0_PIG | SERPINA3-3 | Serpin Family A Member 3 | 24.04±0.10 | 24.49±0.12 | 0.033 |

**^1^**, Abundance is base-2 logarithm transformed. 2, ND, not detected.

**Table S4.** Primer sequence of selected genes analyzed in kidney tissues

| **Symbol** | **Name** | **Primer sequences** | |  |  |  |
| --- | --- | --- | --- | --- | --- | --- |
|  |  | Forward | Reverse | Amplicon length | Efficiency | TM  （F/R） |
| ACE | angiotensin-converting enzyme | GACCACTAGCCAGGTGACAG | TCATCGGACACGAGGTTTGG | 162 | 98.32% | 59.75/60.04 |
| ACE2 | Angiotensin I Converting Enzyme 2 | TGACGTCGAAAAGGCCATCA | CCGGTGAAAATGAGCACGAC | 198 | 102.36% | 59.97/59.83 |
| C3 | Complement C3 | ATCAAATCAGGCTCCGATGA | GGGCTTCTCTGCATTTGATG | 76 | 100.79% | 56.69/57.13 |
| CA | Carbonic anhydrase 2 | AGCACTTAGTGGATGGAGCC | CAGCAGCGCTGGAGTATTTC | 81 | 117.8% | 59.46/59.35 |
| CASP3 | Caspase 3 | ATTGAGACGGACAGTGGGAC | GCTGCACAAAGTGACTGGAT | 152 | 103.7% | 59.39/58.76 |
| CD14 | CD14 Molecule | GGGTTCCTGCTCAGATTCTG | CCCACGACACATTACGGAGT | 165 | 88.8% | 57.96/59.75 |
| FOXP3 | Forkhead box P3 | CATGGAGTACTTCAAGTTCC | AACATGCGTGTGAACCAGTG | 132 | 100.5% | 53.33/59.34 |
| GATA3 | transcription factors GATA3 | ACCCCTTATTAAGCCCAAGC | TCCAGAGAGTCGTCGTTGTG | 92 | 107.20% | 57.55/59.41 |
| HIF1A | Hypoxia Inducible Factor 1 Subunit Alpha | TGTGTTATCTGTCGCTTTGAGTC | TTTCGCTTTCTCTGAGCATTC | 96 | 92.91% | 59.01/56.91 |
| HPRT1 | Hypoxanthine-guanine  phosphoribosyltransferase (REF) | ACACTGGCAAAACAATGCAA | TGCAACCTTGACCATCTTTG | 71 | 94.14% | 57.31/56.53 |
| ICA | Carbonic anhydrase inhibitor | TGTTTACAGACGCCTGCCTT | CAGCTGTCACACTTGGTCCT | 181 | 88.95% | 59.89/59.89 |
| IL10 | Interleukin 10 | GTCCGACTCAACGAAGAAGG | GCCAGGAAGATCAGGCAATA | 73 | 99.6% | 58.3/57.35 |
| IL17 | Interleukin 17 | GCACACGGGCTGCATCAACG | TGCAACCAACAGTGACCCGCA | 149 | 120.3% | 65.16/65.37 |
| IL4 | Interleukin 4 | GTACCAGCAACTTCGTCCAC | CCTTCTCCGTCGTGTTCTCT | 150 | 127.15% | 58.85/59.12 |
| INFr | Interferon-Gamma | AGCTTTGCGTGACTTTGTGT | ATGCTCCTTTGAATGGCCTG | 247 | 125.47% | 58.91/58.52 |
| KIM-1 | Kidney Injury Molecule-1 | ATGTACCCTTGGGTAACCGC | AACGTAGAACATGCCCCTCG | 164 | 99.98% | 59.75/60.11 |
| LRG1 | Leucine Rich Alpha-2-Glycoprotein 1 | TGACCTGCACATCCTTGACC | CAGAAAGCCCTCTTCGAGCA | 133 | 104.7% | 59.96/60.04 |
| LTF | Lactotransferrin | CTGACTGTATCCGGGCCATC | CGTAGATCTCCGCTGCTACC | 114 | 111.03% | 59.97/59.76 |
| LYZ | Lysozyme | TAAAGCATGGGTGGCATGGA | CAGTTTGCAACCCCGAATGT | 73 | 98.74% | 59.67/59.33 |
| NGLA | Neutrophil gelatinase-associated lipocalin | GTTCCAGGGGAAGTGGTACG | GGCTGGAGACTTGGGACAAA | 189 | 101.1% | 60.04/59.89 |
| S100A9 | S100 Calcium Binding Protein A9 | GCCAAACTTTCTCAAGAAGCA | AGTGTCCAGGTCTTCCAGGAT | 70 | 93.52% | 57.28/60.20 |
| SAA | Serum Amyloid A | TGGAGAGCCTACTCGGACAT | CCTTTGGGCAGCATCATAGT | 90 | 94.87% | 59.74/57.94 |
| TBET | Transcription factors T-bet | CTGAGAGTCGCGCTCAACAA | ACCCGGCCACAGTAAATGAC | 121 | 96.89% | 60.66/60.32 |
| TLR4 | Toll like receptor 4 | TGGTGTCCCAGCACTTCATA | CAACTTCTGCAGGACGATGA | 116 | 96.94% | 58.64/57.92 |
| TRL2 | Toll like receptor 2 | CGTGTGCTATGACGCTTTCG | GTACTTGCACCACTCGCTCT | 232 | 107.3% | 59.98/60.04 |

**Table S5.** Primer sequences for liver gene analysis

| **Gene name** | **Gene symbol** | **Sequence (5' to 3')** | **Sequence (5' to 3')** | **Amplicon**  **length** | **Efficiency^1^** |
| --- | --- | --- | --- | --- | --- |
| ACTB | Actin, Beta | F: CTACGTCGCCCTGGACTTC | R: GCAGCTCGTAGCTCTTCTCC | 76 | 96.40% |
| APOA1 (3) | Apolipoprotein A-I | F:GTTCTGGGACAACCTGGAAA | R: GCTGCACCTTCTTCTTCACC | 86 | 97.60% |
| ARG1 (202) | Arginase | F: AATTGGCAAGGTGATGGAAG | R: TCCAGTCCATCCACATCAAA | 90 | 98% |
| B2M (7) | Beta-2-Microglobulin | F: TGAAGCACGTGACTCTCGAT | R: CTCTGTGATGCCGGTTAGTG | 70 | 98.20% |
| BAX(1081) | BCL2 Associated X, Apoptosis Regulator | F: TGCCAGTAAACTGGTGCTCA | R: CGATCTCGAAGGAAGTCCAG | 93 | 95.20% |
| BAX(1082) | BCL2 Associated X, Apoptosis Regulator | F: AACATGGAGCTGCAGAGGAT | R: AGTTGCCGTCAGCAAACATT | 97 | 95.40% |
| BCL2(622) | BCL2, Apoptosis Regulator | F: CCCTGTGGATGACTGAGTACC | R: AACCACACATGCACCTACCC | 83 | 92.90% |
| BCL2(623) | BCL2, Apoptosis Regulator | F: GACTCCCTTCACCGCGAG | R: CTCTCCACACACATGACCCC | 120 | 91% |
| C3 (10) | Complement Component 3 | F:ATCAAATCAGGCTCCGATGA | R: GGGCTTCTCTGCATTTGATG | 76 | 98% |
| C4BPA(151) | Complement Component 4 Binding Protein Alpha | F: GTGAACTGCATGGTTGTGCT | R: TCTTCTGTAGGCAGGGAGTAGC | 82 | NA |
| C5(11) | Complement Component 5 | F: AAGCTGGAGAAGCCGTTGC | R: TTTTCGAGGTTAGCGTTCGT | 82 | 96.90% |
| C5(96) | Complement Component 5 | F: GATGTCTACAAAGCTGGAGAAGC | R: TTTTCGAGGTTAGCGTTCGT | 92 | 97.40% |
| CASP3(383) | Caspase 3 | F: CTGGCAAACCCAAACTTTTC | R: GTCCCACTGTCCGTCTCAAT | 79 | 95.10% |
| CCL5 (121) | C-C Motif Chemokine Ligand 5 | F:CTCCATGGCAGCAGTCGT | R: AAGGCTTCCTCCATCCTAGC | 121 | 97.10% |
| CD14 (8) | CD14 | F: GGGTTCCTGCTCAGATTCTG | R: CCCACGACACATTACGGAGT | 164 | 98.10% |
| CFB(224) | Complement Factor B | F: ATGACCTCAACGCAAAGACC | R: GATCAGGGCCACGTCATAGT | 65 | 96.60% |
| CFD(226) | Complement Factor D | F: AGCTGCAAGGGCGACTC | R: GGGAGCCTGAGGTAACCACT | 73 | 96.00% |
| CFD (227) | Complement Factor D | F: CCTCGGAGCAGCTGTATGT | R: ATGCCATGTAGGGTCTCTCG | 89 | 98.20% |
| CRP(12) | C-Reactive Protein | F: GGTGGGAGACATTGGAGATG | R: GAAGGTCCCACCAGCATAGA | 85 | 96.30% |
| CXCL9(793) | C-X-C Motif Chemokine Ligand 9 | F: AGCAGTGTTGCCTTGCTTTT | R: ATGCAGGAACAACGTCCATT | 92 | 95.40% |
| CXCL10 (111) | Chemokine (C-X-C Motif) Ligand 10 | F:CCCACATGTTGAGATCATTGC | R: GCTTCTCTCTGTGTTCGAGGA | 141 | 98.20% |
| CXCL14(271) | C-X-C Motif Chemokine Ligand 14 | F: GTACCGAGGTCAGGAGCACT | R: TAGACCCTGCGCTTCTCATT | 96 | 97.70% |
| EGF(130) | Epidermal Growth Factor | F: CCAGTAGAAAGGTTGATGTTTTGG | R: TCCAATGACACAGCTGCAAT | 125 | NA |
| FGG(18) | Fibrinogen Gamma Chain | F: CAAAACCAGATAGGATACAAAGTGC | R: CGAATCGTTGAGTCGTGTGT | 103 | 98.30% |
| FGG(77) | Fibrinogen Gamma Chain | F: ATCTTAGATGAGAGATTTGGTAGTTACTG | R: GCCCCTCCAAATTCTGTAGAT | 110 | 98.40% |
| GAPDH (20) | Glyceraldehyde-3-phosphate dehydrogenase | F: ACCCAGAAGACTGTGGATGG | R: AAGCAGGGATGATGTTCTGG | 79 | 98.10% |
| GLUT3/SLC2A3(419) | Solute Carrier Family 2 Member 3 | F: TCCCCTCAGCTGCATTCTAT | R: CCAGAAGACAACGAGGAAGC | 71 | 96.50% |
| GLUT4/SLC2A4(527) | Solute Carrier Family 2 Member 4 | F: ACTGTGGCTCTGCTTCTGCT | R: CTGGGCCAATCTCAAAGAAG | 97 | 97.50% |
| HPX(23) | Hemopexin | F: ACCTGGTCTTGACTGCACTG | R: GGTGTCCAGACGCCAGTAGT | 83 | 97.30% |
| HIF1A(1073) | Hypoxia Inducible Factor 1 Alpha Subunit | F: TTACAGCAGCCAGATGATCG | R: TCTTTTGCTCCGTTCCATTC | 97 |  |
| HIF1A(1074) | Hypoxia Inducible Factor 1 Alpha Subunit | F: GAATGGAACGGAGCAAAAGA | R: TGATTGCCCCAGGAGTCTAC | 70 | 95.90% |
| HMOX1 (465) | Heme Oxygenase 1 | F:AAGACCGCCTTCCTGCT | R: CCTTAGTGTCCTGGGTCAGC | 70 | 97.50% |
| HP(21) | Haptoglobin | F: ACAGATGCCACAGATGACAGC | R: CGTGCGCAGTTTGTAGTAGG | 105 | 98.20% |
| IFNA (49) | Interferon alpha 1 | F:ATCGTCAGGGCAGAAGTCAT | R: CCAGGTGTCTGTCACTCCTTC | 86 | 94.40% |
| NFKBIA(131) | NFKB Inhibitor Alpha | F: GAGGATGAGCTGCCCTATGAC | R: CCATGGTCTTTTAGACACTTTCC | 85 | 98.40% |
| IL15(292) | Interleukin 15 | F: CGTCATTTTGCAAGAGTCCA | R: TGGACGATAAACTGCTGTTTGC | 86 | 92.70% |
| IL18 (234) | Interleukin 18 | F: CAATTGCATCAGCTTTGTGG | R: TCCAGGTCCTCATCGTTTTC | 78 | 95.30% |
| IL1RN (142) | Interleukin 1 Receptor Antagonist | F:TGCCTGTCCTGTGTCAAGTC | R: GTCCTGCTCGCTGTTCTTTC | 90 | 98.40% |
| IL6ST (277) | Interleukin 6 Signal Transducer | F: TGAAGGTGGAAAGGATGGTC | R: GGGTTGTCAACAGGAATGCT | 110 | 97.30% |
| IL6ST(278) | Interleukin 6 Signal Transducer | F: ATCCCTGCCTGTGATTTTCA | R: TGGAGCAGTCCATTCTACCC | 93 | 97.60% |
| IRAK4(1077) | Interleukin 1 Receptor Associated Kinase 4 | F: CGACTGTCTTGCTTGGATGA | R: ACTGAGGCCATTAGCTGCAC | 84 | 97.40% |
| ITGAM(577) | Integrin Subunit Alpha M | F: TAACCCTCTCCCACTCATCG | R: AAGCCGAGCTTGTACAGTCC | 87 | NA |
| ITIH4(56) | Inter-Alpha-Trypsin Inhibitor Heavy Chain Family Member 4 | F: ATGACAGCAAGCGAACAGTG | R: GGGGATCCCTCTTGGTAATC | 85 | 97.90% |
| LBP(82) | Lipopolysaccharide Binding Protein | F: CCCAAGGTCAATGATAAGTTGG | R: ATCTGGAGAACAGGGTCGTG | 83 | 92.30% |
| LBP(83) | Lipopolysaccharide Binding Protein | F: ATTCAATGTGGAGCTGTT | R: GGAAGCCTTCTGCCAACT | 92 | 98.00% |
| MMP2 (332) | Matrix Metallopeptidase 2 | F:TCGCTGGAGATAAGTTCTGGAG | R: GGCGTCTGCAATGAGCTT | 80 | 98.80% |
| MMP8(857) | Matrix Metallopeptidase 8 | F: GGCTGCCTATGAGGATTCTG | R: TGAATGTCATAGCCGCTCAG | 84 | NA |
| MYD88(179) | Myeloid Differentiation Primary Response 88 | F: CCAGACTAAGTTTGCACTCAGC | R: AGGATGCTGGGGAACTCTTT | 99 | 89.00% |
| NFKB1 (612) | Nuclear Factor Kappa B Subunit 1 | F: CCCTGTGAAGACCACCTCTC | R: ATCCCGGAGCTCGTCTATTT | 82 | 95.80% |
| NOD1(366) | Nucleotide Binding Oligomerization Domain Containing 1 | F: CAGTGGGGTGAAGGTGCTAT | R: TACCTGGCTCCGACATCAGT | 99 | 95.90% |
| ORM(5) | Orosomucoid 1 | F: AGTCCTGAGCCTCCTTCCTC | R: GCCGAGCCGATATAATACCA | 123 | 97.90% |
| PPIA (154) | peptidylprolyl isomerase A (cyclophilin A) | F: CAAGACTGAGTGGTTGGATGG | R: TGTCCACAGTCAGCAATGGT | 138 | 98.80% |
| RPL13A | Ribosomal protein L13a | F: ATTGTGGCCAAGCAGGTACT | R: AATTGCCAGAAATGTTGATGC | 76 | 98.00% |
| S100A8(932) | S100 Calcium Binding Protein A8 | F: AATTACCACGCCATCTACGC | R: CAGGTTTCTGCGTCCTTTTT | 89 | 97.60% |
| STAT1(122) | Signal Transducer And Activator Of Transcription 1 | F: CCTTGCAGAATAGAGAACATGATAC | R: CCTTTCTCTTGTTGTCAAGCATT | 108 | 97.70% |
| SAA (243) | Serum Amyloid A | F: CAGAGATGGGCATCATTCCT | R: TGGCATCGCTGATCACTTTA | 184 | 98.20% |
| SAA (60) | Serum Amyloid A | F: TGGAGAGCCTACTCGGACAT | R: CCTTTGGGCAGCATCATAGT | 90 | 95.10% |
| TBP | TATA box binding protein | F: ACGTTCGGTTTAGGTTGCAG | R: CAGGAACGCTCTGGAGTTCT | 96 | 97.50% |
| YWHAE (156) | Tyrosine 3-monooxygenase/tryptophan 5-monooxygenase | F: GCTGCTGGTGATGATAAGAAGG | R: AGTTAAGGGCCAGACCCAAT | 124 | 98.10% |
| TLR2(160) | Toll like receptor 2 | F: CGGAGGTTGCATATTCCACAG | R: TGTGAAAGGGAACAGGGAAC | 128 | 94.00% |
| TLR4(235) | Toll like receptor 4 | F: TGGTGTCCCAGCACTTCATA | R: CAACTTCTGCAGGACGATGA | 116 | 95.30% |
| TNF (74) | Tumor Necrosis Factor alpha | F:CCCCCAGAAGGAAGAGTTTC | R: CGGGCTTATCTGAGGTTTGA | 92 | 97.30% |
| TOLLIP(541) | Toll Interacting Protein | F: GACCCGTACTGCCGACTG | R: ATCACCTTATTCCAGCGCGG | 92 | 97.90% |
| TF(64) | Transferrin | F: CTCAACCTCAAAACTCCTGGAA | R: CCGTCTCCATCAGGTGGTA | 82 | 98.10% |
| TTR(66) | Transthyretin | F: TGCTGGTGAATCCAAGTGTC | R: CACTTTCACGCCTACGTTCA | 85 | 97.90% |

1, NA, data and efficiency are not available.

**Table S6.** Liver gene expressions

| **Day 1** | | | |  | **Day 5** | | | |
| --- | --- | --- | --- | --- | --- | --- | --- | --- |
| **Gene name** | **Fold change^1^**  **(LPS to control)** | **STD** | **P-Value** |  | **Gene name** | **Fold change^1^**  **(LPS to control)** | **STD** | **P-Value** |
| APOA1(3) | 0.9715 | 0.3921 | 0.6212 |  | APOA1(3) | 0.8841 | 0.2236 | 0.2907 |
| ARG(202) | 1.0673 | 0.5040 | 0.4963 |  | ARG(202) | 0.8516 | 0.2835 | 0.5735 |
| B2M(7) | 1.6005 | 0.5169 | 0.0031 |  | B2M(7) | 0.6422 | 0.2234 | 0.3039 |
| B-ACTIN(6) | 0.9598 | 0.3410 | 0.9695 |  | B-ACTIN(6) | 0.8712 | 0.2634 | 0.2629 |
| BAX(1081) | 0.9123 | 0.3270 | 0.7308 |  | BAX(1081) | 1.0810 | 0.3463 | 0.6237 |
| BAX(1082) | 1.1026 | 0.1902 | 0.2605 |  | BAX(1082) | 0.9761 | 0.2888 | 0.9342 |
| BCL2(622) | 1.4068 | 1.2602 | 0.5304 |  | BCL2(622) | 1.0223 | 0.4642 | 0.6417 |
| BCL2(623) | 1.1532 | 0.6581 | 0.9869 |  | BCL2(623) | 0.9591 | 0.5062 | 0.5503 |
| C3(10) | 2.3133 | 0.6278 | 0.0000 |  | C3(10) | 0.6189 | 0.3203 | 0.0840 |
| C4BPA(151) | 1.3017 | 0.8258 | 0.7225 |  | C4BPA(151) | 1.6197 | 0.8309 | 0.0550 |
| C5(11) | 1.5303 | 0.6154 | 0.1227 |  | C5(11) | 0.7203 | 0.3670 | 0.3780 |
| C5(96) | 1.4735 | 0.5894 | 0.1944 |  | C5(96) | 0.7560 | 0.4050 | 0.3385 |
| C5(96) | 1.4631 | 0.5791 | 0.2383 |  | C5(96) | 0.7595 | 0.3636 | 0.3954 |
| CASP3(383) | 1.1386 | 0.5920 | 0.7360 |  | CASP3(383) | 0.9311 | 0.2671 | 0.5464 |
| CCL5(121) | 1.5531 | 1.1030 | 0.2235 |  | CCL5(121) | 0.8683 | 0.3299 | 0.3776 |
| CD14(8) | 1.2604 | 0.5271 | 0.1888 |  | CD14(8) | 0.8508 | 0.2870 | 0.8538 |
| CD14(8) | 1.2299 | 0.3905 | 0.2396 |  | CD14(8) | 0.8926 | 0.2928 | 0.9307 |
| CFB(224) | 0.8998 | 0.2137 | 0.1873 |  | CFB(224) | 1.0256 | 0.1513 | 0.6581 |
| CFD(226) | 0.9747 | 0.3259 | 0.6307 |  | CFD(226) | 0.9449 | 0.1010 | 0.6200 |
| CFD(227) | 0.9685 | 0.3802 | 0.5814 |  | CFD(227) | 0.9475 | 0.4021 | 0.7907 |
| CRP(12) | 1.1796 | 0.7164 | 0.3786 |  | CRP(12) | 0.2992 | 0.3145 | 0.0611 |
| CXCL 9(793) | 1.3060 | 1.1324 | 0.5668 |  | CXCL 9(793) | 0.3345 | 0.1968 | 0.1933 |
| CXCL10(111) | 2.7764 | 2.5262 | 0.0202 |  | CXCL10(111) | 0.4518 | 0.4506 | 0.3629 |
| CXCL14(271) | 1.1160 | 0.5127 | 0.4705 |  | CXCL14(271) | 0.9091 | 0.7287 | 0.6035 |
| EGF(130) | 1.0122 | 0.7186 | 0.5318 |  | EGF(130) | 1.3263 | 1.4090 | 0.8467 |
| FIB(18) | 1.0992 | 0.3070 | 0.4714 |  | FIB(18) | 0.7891 | 0.1929 | 0.3069 |
| FIB(77) | 1.1834 | 0.1906 | 0.0999 |  | FIB(77) | 0.7228 | 0.2343 | 0.0173 |
| GAPDH(20) | 1.0498 | 0.2920 | 0.7171 |  | GAPDH(20) | 1.0146 | 0.2439 | 0.8055 |
| GLUT3(419) | 1.6407 | 1.9717 | 0.6497 |  | GLUT3(419) | 0.9511 | 0.4446 | 0.9699 |
| GLUT4(527) | 0.8708 | 0.7308 | 0.1654 |  | GLUT4(527) | 0.4400 | 0.2690 | 0.1845 |
| HEM(23) | 1.3093 | 0.3710 | 0.0865 |  | HEM(23) | 0.8791 | 0.3029 | 0.3339 |
| HIF1A(1073) | 1.0237 | 0.2182 | 0.8162 |  | HIF1A(1073) | 1.0402 | 0.2303 | 0.7036 |
| HIF1A(1074) | 1.1414 | 0.3034 | 0.2835 |  | HIF1A(1074) | 1.0169 | 0.2617 | 0.9503 |
| HMOX1(465) | 1.0008 | 0.1608 | 0.7255 |  | HMOX1(465) | 0.9864 | 0.3626 | 0.7959 |
| HP(21) | 1.5277 | 0.8638 | 0.2233 |  | HP(21) | 0.7669 | 0.4346 | 0.6918 |
| IFN?(49) | 1.5283 | 1.8066 | 0.7929 |  | IFN?(49) | 1.1239 | 0.8799 | 0.7245 |
| IKBA(131) | 1.0105 | 0.4633 | 0.8479 |  | IKBA(131) | 0.4784 | 0.1663 | 0.1903 |
| IL15(292) | 1.6141 | 1.1565 | 0.2268 |  | IL15(292) | 0.6328 | 0.2820 | 0.3631 |
| IL18(234) | 1.6856 | 1.2248 | 0.2476 |  | IL18(234) | 0.6011 | 0.3477 | 0.4276 |
| IL1RN(142) | 0.9787 | 0.3329 | 0.7748 |  | IL1RN(142) | 0.9273 | 0.8263 | 0.9253 |
| IL6ST(227) | 1.0776 | 0.3482 | 0.7029 |  | IL6ST(227) | 1.0537 | 0.3264 | 0.7718 |
| IL6ST(278) | 1.1539 | 0.4997 | 0.5776 |  | IL6ST(278) | 0.8631 | 0.2004 | 0.2374 |
| IRAK4(1077) | 1.0321 | 0.3801 | 0.8231 |  | IRAK4(1077) | 0.9525 | 0.2011 | 0.8566 |
| IRAK4(1077) | 1.0610 | 0.4156 | > 0.99 |  | IRAK4(1077) | 0.9998 | 0.2296 | 0.9498 |
| ITGAM(577) | 1.1777 | 1.1177 | 0.7734 |  | ITGAM(577) | 1.1107 | 0.7152 | 0.4009 |
| ITIH4(56) | 1.6381 | 0.5770 | 0.0100 |  | ITIH4(56) | 0.3861 | 0.2204 | 0.0476 |
| LPSBP(82) | 1.4267 | 1.6626 | 0.6061 |  | LPSBP(82) | 0.5102 | 0.2118 | 0.5222 |
| LPSBP(83) | 1.4186 | 0.5552 | 0.0625 |  | LPSBP(83) | 0.6593 | 0.3155 | 0.2592 |
| MMP2() | 1.0834 | 0.5034 | 0.9289 |  | MMP2() | 1.1152 | 0.3050 | 0.3586 |
| MMP8(857) | 1.5443 | 0.8609 | 0.1705 |  | MMP8(857) | 0.8770 | 0.7838 | 0.3443 |
| MYD88 (179) | 1.3522 | 0.6679 | 0.1748 |  | MYD88 (179) | 0.8946 | 0.3628 | 0.7287 |
| NF-KB(612) | 1.3441 | 0.6233 | 0.1196 |  | NF-KB(612) | 0.7310 | 0.3085 | 0.1927 |
| NOD1(366) | 1.2313 | 0.6866 | 0.4087 |  | NOD1(366) | 1.2108 | 0.6442 | 0.5475 |
| ORM(5) | 0.9744 | 0.2911 | 0.6673 |  | ORM(5) | 0.8821 | 0.2730 | 0.5431 |
| S100A8(932) | 1.9671 | 0.7986 | 0.0087 |  | S100A8(932) | 0.3837 | 0.1985 | 0.0910 |
| SAA (243) | 10.5225 | 11.2679 | 0.0001 |  | SAA (243) | 1.0335 | 1.7752 | 0.7368 |
| SAA(60) | 8.1064 | 8.5822 | 0.0001 |  | SAA(60) | 0.5458 | 0.7375 | 0.3162 |
| STAT1(122) | 1.1972 | 0.4644 | 0.4746 |  | STAT1(122) | 1.1418 | 0.5515 | 0.6248 |
| TLR2(160) | 1.8903 | 1.1930 | 0.0693 |  | TLR2(160) | 0.9383 | 0.4255 | 0.6640 |
| TLR4(235) | 1.5726 | 0.6495 | 0.0155 |  | TLR4(235) | 0.9349 | 0.3228 | 0.4414 |
| TNF(74) | 1.3563 | 1.1637 | 0.9167 |  | TNF(74) | 0.3421 | 0.2478 | 0.5780 |
| TOLLIP(541) | 0.9237 | 0.4950 | 0.5529 |  | TOLLIP(541) | 0.8552 | 0.4450 | 0.8209 |
| TRF(64) | 0.9130 | 0.3436 | 0.9869 |  | TRF(64) | 1.0067 | 0.8666 | 0.5417 |
| TTR(66) | 0.6232 | 0.2063 | 0.0322 |  | TTR(66) | 0.8337 | 0.3872 | 0.3448 |

1, Fold change iscalculated base-2 logarithm transformed data.

1. Nguyen DN, Thymann T, Goericke-Pesch SK, Ren S, Wei W, Skovgaard K, Damborg P, Brunse A, van Gorp C, Kramer BW, Wolfs TG, Sangild PT (2018) Prenatal Intra-Amniotic Endotoxin Induces Fetal Gut and Lung Immune Responses and Postnatal Systemic Inflammation in Preterm Pigs. The American journal of pathology 188 (11):2629-2643. doi:10.1016/j.ajpath.2018.07.020
